# Probiotic Potential and Enhanced Adhesion of Fermented Foods-Isolated Lactic Acid Bacteria to Intestinal Epithelial Caco-2 and HT-29 Cells

**DOI:** 10.3390/microorganisms13010032

**Published:** 2024-12-27

**Authors:** Eun Ah Sim, Seon-Young Kim, SangNam Kim, Eun-Gyung Mun

**Affiliations:** Jeonju AgroBio-Materials Institute (JAMI), Jeonju-si 54810, Republic of Korea; sea0401@jami.re.kr (E.A.S.);

**Keywords:** lactic acid bacteria (LAB), probiotics, adhesion capacity

## Abstract

This study evaluated the probiotic potential of lactic acid bacteria (LAB) isolated from fermented milk and soymilk products purchased from local markets. The LAB strains were assessed for acid and bile resistance, antibiotic resistance, and adhesion to human intestinal epithelial models. *Streptococcus thermophilus* (JAMI_LB_02) and *Lactiplantibacillus plantarum* (JAMI_LB_05) showed the highest survival rates in artificial gastric and bile juices, at 87.17 ± 0.02% and 96.71 ± 4.10%, respectively, with all strains (except JAMI_LB_03) demonstrating antibiotic resistance. Adhesion ability indicated the superior performance of JAMI_LB_02 and JAMI_LB_05 compared to standard strains. JAMI_LB_02 adhered to Caco-2 cells at 2.10 ± 0.94% and to HT-29 cells at 3.32 ± 0.38%, exceeding standard strains (1.06 ± 0.13% and 1.89 ± 0.58%). JAMI_LB_05 achieved the highest rates at 5.62 ± 1.33% on Caco-2 and 5.76 ± 0.46% on HT-29 cells. Their combination (JAMI_LB_02 + JAMI_LB_05) significantly enhanced adhesion to 18.57 ± 5.49% on Caco-2 and 21.67 ± 8.19% on HT-29 cells, demonstrating strong synergy. These findings highlight the probiotic potential of the isolated LAB strains, particularly in mixed formulations, which may improve intestinal survival, adaptability, and efficacy. Further in vivo studies are warranted to validate their clinical applications and optimize strain combinations for human health benefits.

## 1. Introduction

Probiotics are living microorganisms that confer health benefits to the host when administered in adequate amounts. They enhance the balance of intestinal microbiota, support digestive health, strengthen the immune system, and help reduce cholesterol levels [1,2]. To qualify as probiotics, strains must be recognized as ‘generally regarded as safe’ (GRAS). Primary probiotic strains used in human applications include the species *Lactobacillus*, *Bifidobacterium*, *Enterococcus*, *Streptococcus*, and *Lactococcus* [3,4,5]. Most commercially utilized probiotics are animal-derived lactic acid bacteria (LAB), commonly isolated from the human gut or dairy products. LAB have gained prominence as functional food components due to their roles in promoting digestion and alleviating conditions such as irritable bowel syndrome and inflammatory bowel disease through microbiome modulation [6].

For probiotics to exert their physiological effects within the human body, they must demonstrate high viability under harsh conditions, such as exposure to gastric and bile juices. Additionally, they must adhere effectively to colorectal epithelial cells and possess the ability to produce antimicrobial substances that suppress harmful bacteria [7]. These properties ensure that probiotics survive digestion, reach the intestine alive, and exhibit their functional benefits. Strains capable of colonizing epithelial cells in the intestinal mucosa can promote mucin production, reduce intestinal permeability, and protect intestinal epithelial cells. This, in turn, inhibits the adhesion of pathogenic microorganisms and the penetration of toxic substances, thereby strengthening the host’s defense mechanisms [8]. However, the adhesion capability of LAB to intestinal epithelial cells varies across strains, and the health benefits associated with probiotics are often strain-specific [9]. Consequently, evaluating factors such as survival rate and adhesion ability is crucial for assessing the probiotic activity of individual strains.

Recent studies have highlighted health benefits associated with LAB. For example, *Lactiplantibacillus plantarum* can suppress skin cancer [10], enhance immunity via NF-κB pathway regulation [11], and lower serum cholesterol levels [12]. Similarly, *Lacticaseibacillus paracasei* demonstrates strong adaptability to diverse environments and immune system-strengthening properties [13]. *Streptococcus thermophilus* aids lactose digestion and alleviates gastrointestinal symptoms through lactase production [14]. These findings underscore the diverse health functionalities of LAB, which depend on the strain and its specific characteristics. Notably, combining multiple LAB strains may yield greater health benefits than using individual strains, as mixed cultures can target a broader range of conditions [15]. For instance, Kim et al. [16] demonstrated that a mixture of three *Bifidobacterium* and *Lactobacillus* strains significantly reduced total cholesterol, triglycerides, and low-density lipoprotein cholesterol levels in a hypercholesterolemic rat model. Similarly, Fijan et al. [17] reported that mixtures of *Lactobacilli*, *Bifidobacteria*, and *Enterococci* strains exhibited stronger antagonistic activity against *Escherichia coli* compared to single strains.

Despite extensive research on the health effects of single LAB strains, studies examining the benefits of LAB mixtures remain limited. This study aimed to evaluate the probiotic activity of LAB strains isolated from fermented milk and beverages by assessing their acid and bile resistance, antibiotic resistance, and adhesion to intestinal epithelial cells using Caco-2 and HT-29 cell models. Additionally, the study explored the effects of LAB mixtures on probiotic activity, highlighting their potential as functional materials based on their physiological properties.

## 2. Materials and Methods

### 2.1. Strain Isolation

Strains were isolated from concentrated and filtered fermented milk and from fermented beverages purchased from local markets (Gyeonggi-do, Republic of Korea). Samples were mixed in MRS broth and shaken and incubated at 37 °C. Afterwards, five colonies representing the majority of strains with various shapes for each sample were inoculated onto PCA with BCP agar, and one colony with a yellow ring was selected. A single colony was isolated by streaking on MRS agar three or more times. The isolates were stored at −40 °C in MRS broth containing 25% glycerol and used in experiments.

### 2.2. Strain Identification

The isolated strain was identified using the 16S rRNA gene base sequence. The gene was amplified using universal primers 27F (5′-AGAGTTTTGATCCTGGCTCAG-3′) and 1492R (5′-GGTTACCTTGTTACGACTT-3′), the PCR product was purified, and the base sequence was decoded. Using BLSATN search [18] and the SeqMatch program of the Ribosomal Database Project (RDPm ver. 11), the base sequence of the decoded strain and the 16S rRNA gene base sequence of the standard strain with high sequence identity were obtained, and the interaction between base sequences was obtained. The CLUSTAL W program [19] was used for comparison. For phylogenetic analysis, the 16S rRNA gene base sequences of the strains were aligned and corrected to minimize the gap, and then the maximum likelihood method based on the Tamura–Nei model [20] was used. The statistical confidence for the molecules in each phylogenetic tree was calculated by setting the bootstrap to 1000 times, and a phylogenetic analysis tree was created using the MEGA program 11 [21].

### 2.3. Preparation of Strain Culture

The LAB were activated by subculture twice to MRS agar and cultured in MRS broth at 37 °C for 24 h. The culture medium was prepared by adjusting the absorbance to 1.0 at 600 nm of a spectrophotometer (UV-1601, Shimadzu, Kyoto, Japan), and the pellet was recovered by centrifugation (8000× *g*, 5 min). Subsequently, it was suspended using phosphate-buffered saline (PBS, Gibco, Waltham, MA, USA) for further use.

### 2.4. Survival of Probiotics in the Gastric and Intestinal Juice

The survival of probiotics in the gastric and intestinal juice was determined following the method described by Talib et al. [22] with some modifications. Artificial gastric juice was prepared by dissolving pepsin in MRS broth to a concentration of 1 mg/mL, and its pH was adjusted to 2.5 using HCl. The artificial bile juice was prepared by adding 3% bile salts to MRS broth. The supernatant was removed by centrifugation of the strain culture medium with adjusted absorbance, and the cells were recovered and suspended in artificial gastric juice and bile juice. Subsequently, it was cultured at 37 °C for 2 h, and the serial dilution was streaked onto MRS agar. The survival rates within the artificial gastric juice and bile juice were calculated using the following equation:

$$Gastric\;and\;intestinal\;juice\;survival\;of\;probiotics\;(\%)=\frac{Viable\;bacteria\;count\;in\;gastric\;juice\;and\;bile\;juice}{Viable\;bacteria\;cell\;count\;in\;MRS\;broth}\times 100$$

### 2.5. Antibiotic Resistance in Probiotics

Antibiotic resistance was determined by testing the minimum inhibitory concentration (MIC) following the EFSA guidelines for testing antimicrobial susceptibility [23]. Antibiotics, including ampicillin, tetracycline, erythromycin, and streptomycin, were tested based on the cut-off values. The LAB were sub-cultured twice in MRS agar and inoculated into MRS broth. The preculture medium was coated on a sterilized cotton swab after adjusting the number of bacteria in the strain to 5 × 10^8^ CFU/mL and streaked onto MRS agar. A paper disk (8 mm) was put on MRS agar that was inoculated with the bacterial suspension, loaded with 20 µL of antibiotics, and cultured at 37 °C for 24 h to measure the MIC that produced an inhibitory zone around the disk.

### 2.6. In Vitro Adhesion Assay

The adhesion of indigenous LAB isolates was measured following the method described by Sharma and Kanwar [24]. When Caco-2 and HT-29 cells formed a monolayer, they were washed twice with PBS, and Dulbecco’s modified eagle medium (DMEM) without fetal bovine serum containing antibiotics was added and incubated for 30 min. Subsequently, 1 mL of LAB culture medium with a controlled number of bacteria was centrifuged (8000× *g*, 5 min), suspended in DMEM, and then added to the cell. The cells were incubated for 2 h, washed five times with PBS, and fixed with 1 mL of methanol for 10 min. The Giemsa stain solution was diluted in PBS at a ratio of 1:20 and stained at room temperature for 20 min. Thereafter, it was washed more than five times with distilled water and observed by microscope.

### 2.7. Statistical Analyses

The data are expressed as the mean ± the standard deviation (SD) of three independent experiments. Statistical analysis was performed using SPSS statistics 12.0 (SPSS Inc. Chicago, IL, USA). Comparisons among groups were analyzed by one-way ANOVA followed by post-hoc analysis using Duncan’s multiple range test with the significance level set at *p* < 0.05.

## 3. Results

### 3.1. Identification of Strain

**Figure 1:**
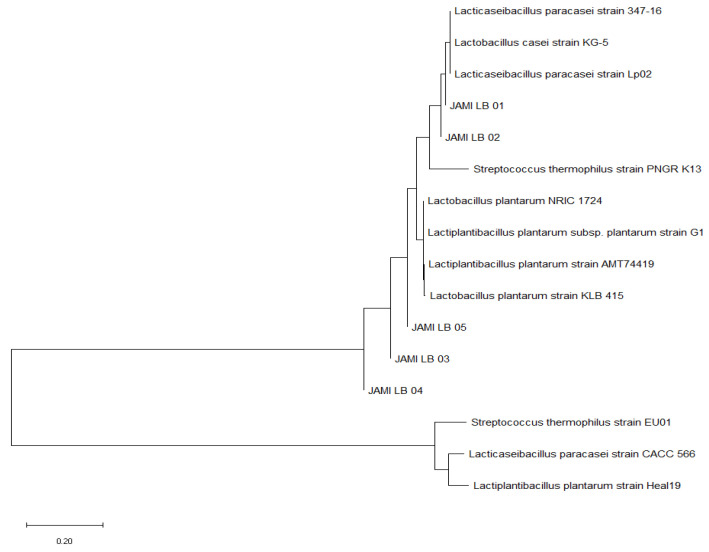
Phylogenetic tree of bacterial strains isolated from fermented milk and beverages constructed using the maximum likelihood method based on 16S rRNA sequences.

Bacteria isolated from various fermented milk and beverage samples were identified based on their 16S rRNA gene sequences. A standard strain with the highest match rate to the base sequence was selected, and a phylogenetic tree was constructed using the MEGA program (Figure 1). This analysis identified one strain of *Lacticaseibacillus paracasei*, one strain of *Streptococcus thermophilus*, and three strains of *Lactiplantibacillus plantarum*. The similarity of 16S sequences to that of reference strains in GenBank is shown in Table 1. Strain JAMI_LB_01, isolated from fermented soy milk, exhibited 99.80% nucleotide sequence identity with *L. paracasei*. Strain JAMI_LB_02, obtained from fermented soy milk, showed 99.73% similarity with *S. thermophilus*. Strains JAMI_LB_03, isolated from filtered fermented milk, and JAMI_LB_04 and JAMI_LB_05, isolated from fermented milk, demonstrated 99.86%, 99.93%, and 99.93% similarity to *L. plantarum*, respectively.

### 3.2. Survival of LAB in an Acidic Environment

The survival rates of the five LAB strains in artificial gastric juice were evaluated by culturing the strains for 2 h at pH 2.5, adjusted with HCl. The results of the acid resistance experiments of the LABs are shown in Table 2. Survival rates varied depending on the strain. The *L. plantarum* strains (JAMI_LB_03, JAMI_LB_04, and JAMI_LB_05) demonstrated high survival rates under acidic conditions, with survival rates of 90.70 ± 4.45%, 95.73 ± 3.52%, and 96.71 ± 4.10%, respectively. These rates were significantly higher compared to the other strains. Strain JAMI_LB_01 (*L. paracasei*) exhibited a survival rate of 57.67 ± 0.03%, while JAMI_LB_02 (*S. thermophilus*) showed the lowest survival rate at 42.31 ± 1.95%.

When strains were mixed, the survival rates approximated the average of the individual strains. Mixtures JAMI_LB_01 + JAMI_LB_03, JAMI_LB_01 + JAMI_LB_05, JAMI_LB_02 + JAMI_LB_03, and JAMI_LB_02 + JAMI_LB_05 exhibited survival rates of 77.76 ± 2.06%, 71.43 ± 7.11%, 73.62 ± 7.65%, and 73.41 ± 7.44%, respectively.

### 3.3. Survival of LAB in the Bile Salt Environment

Survival rates in artificial intestinal juice, tested by inoculating the strains into a medium containing 3% bile salts and culturing for 2 h, also varied. The results of the bile salt resistance experiments of the LABs are shown in Table 2. Strain JAMI_LB_02 exhibited the highest survival rate at 87.17 ± 0.02%, followed by strains JAMI_LB_03, JAMI_LB_04, and JAMI_LB_05, all showing survival rates exceeding 70%. Strain JAMI_LB_01 showed a comparatively lower survival rate of 50.71 ± 8.52%. Mixtures followed a similar trend to gastric juice tests, with JAMI_LB_01 + JAMI_LB_03 and JAMI_LB_02 + JAMI_LB_03 exhibiting survival rates of 70.45 ± 3.55% and 77.21 ± 2.23%, respectively, which were significantly higher than those of other mixtures.

### 3.4. Antibiotic Resistance

Probiotic LAB require resistance against antibiotics, including those that inhibit cell wall, protein, and nucleic acid synthesis, as well as antibiotics that compete with metabolic processes [25]. An experiment was conducted to evaluate the antibiotic resistance of LAB strains when exposed to digestive juices and antibiotics. The MIC was determined for five antibiotics following EFSA guidelines, and cut-off values were established.

The results of antibiotic resistance are summarized in Table 3. Resistance profiles varied among strains. The JAMI_LB_01, JAMI_LB_04, and JAMI_LB_05 strains exhibited high resistance to all tested antibiotics. JAMI_LB_02 was resistant to kanamycin but susceptible to other antibiotics, whereas JAMI_LB_03 was resistant to all antibiotics except ampicillin.

### 3.5. Adhesion Ability to Caco-2 and HT-29 Cell Lines Under In Vitro Conditions

**Figure 2:**
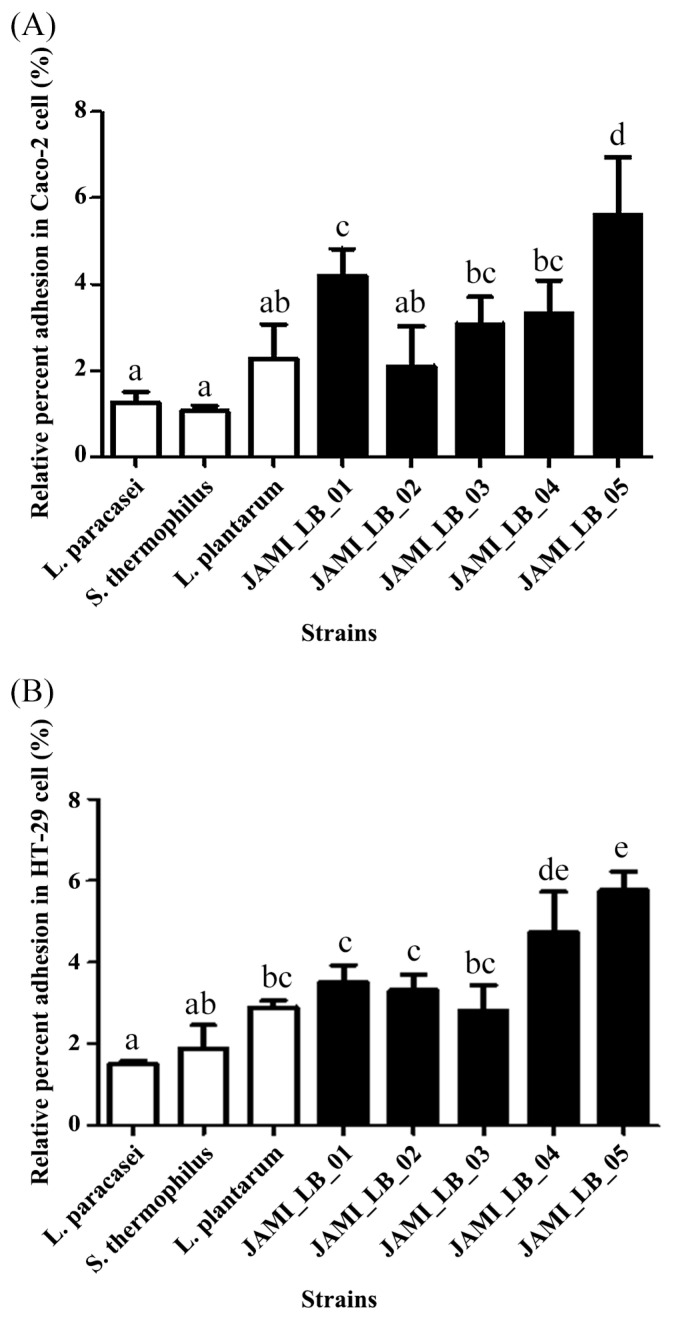
Adhesion of individual strains to (**A**) Caco-2 and (**B**) HT-29 cells. Results are expressed as means ± SD of triplicate determinations. Different lowercase letters (e.g., a, b, c, d, e) within the same line denote significant differences, as determined by Duncan’s multiple range test (*p* < 0.05).

The LAB were adjusted to a concentration of 10^8^ CFU/mL, and Caco-2 and HT-29 cells were treated to evaluate adhesion ability. The quantification of LAB attached to Caco-2 cells is shown in Figure 2A. All strains isolated from fermented milk and beverages exhibited higher adherence to Caco-2 cells compared to their respective standard strains. The adherence capacity of JAMI_LB_01 was 4.20 ± 0.62%, significantly exceeding that of the standard strain *L. paracasei* (1.26 ± 0.25%). Similarly, JAMI_LB_02 demonstrated an adherence capacity of 2.10 ± 0.94%, surpassing *S. thermophilus* (1.06 ± 0.13%). The adherence rates for JAMI_LB_03, JAMI_LB_04, and JAMI_LB_05 were also significantly higher than those of *L. plantarum* (2.28 ± 0.79%), achieving rates of 3.08 ± 0.63%, 3.33 ± 0.76%, and 5.62 ± 1.33%, respectively.

**Figure 3:**
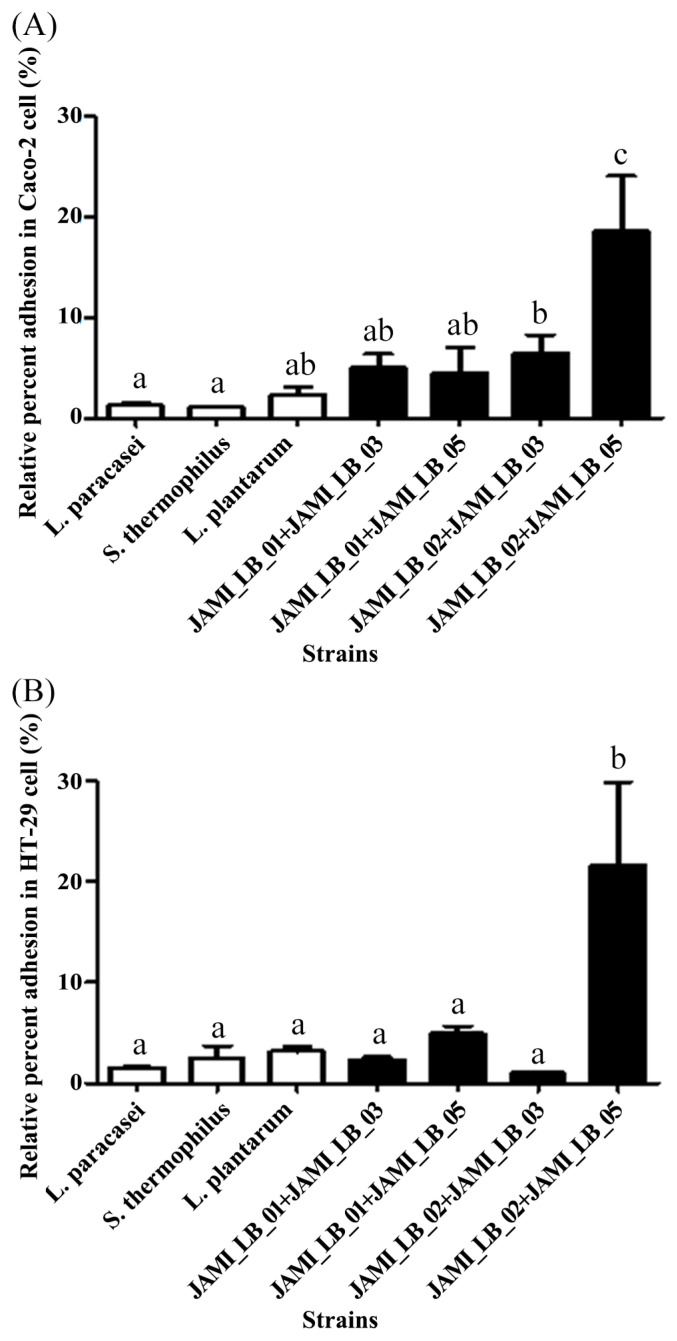
Adhesion ability of mixed strains to (**A**) Caco-2 and (**B**) HT-29 cells. Results are expressed as means ± SD of triplicate experiments. Different lowercase letters (e.g., a, b, c) within the same line denote significant differences, as determined by Duncan’s multiple range test (*p* < 0.05).

Adhesion to HT-29 cells was quantified in Figure 2B. Except for JAMI_LB_03, all strains demonstrated significantly higher adhesion rates than their respective standard strains. JAMI_LB_01 and JAMI_LB_02 showed adhesion capacities of 3.51 ± 0.43% and 3.32 ± 0.38%, respectively, compared to their standard strains (1.52 ± 0.07% and 1.89 ± 0.58%). Notably, JAMI_LB_04 and JAMI_LB_05 exhibited the highest adhesion levels at 4.73 ± 1.00% and 5.76 ± 0.46%, respectively. When LAB strains were applied as mixtures rather than individually, adhesion ability significantly improved. Figure 3 illustrates the results of LAB strain mixtures on Caco-2 cells (A) and HT-29 cells (B). The adhesion rate of the JAMI_LB_02 + JAMI_LB_03 combination to Caco-2 cells was 6.43 ± 1.80%, indicating a substantial increase. In the JAMI_LB_02 + JAMI_LB_05 combination, adhesion reached 18.57 ± 5.49%, more than triple the rate observed with single-strain treatments. Similarly, in HT-29 cells, the JAMI_LB_02 + JAMI_LB_05 mixture achieved a rate of 21.67 ± 8.19%, over four times higher than sitngle-strain treatments, highlighting the superior adhesive potential of the mixed strains.

**Figure 4:**
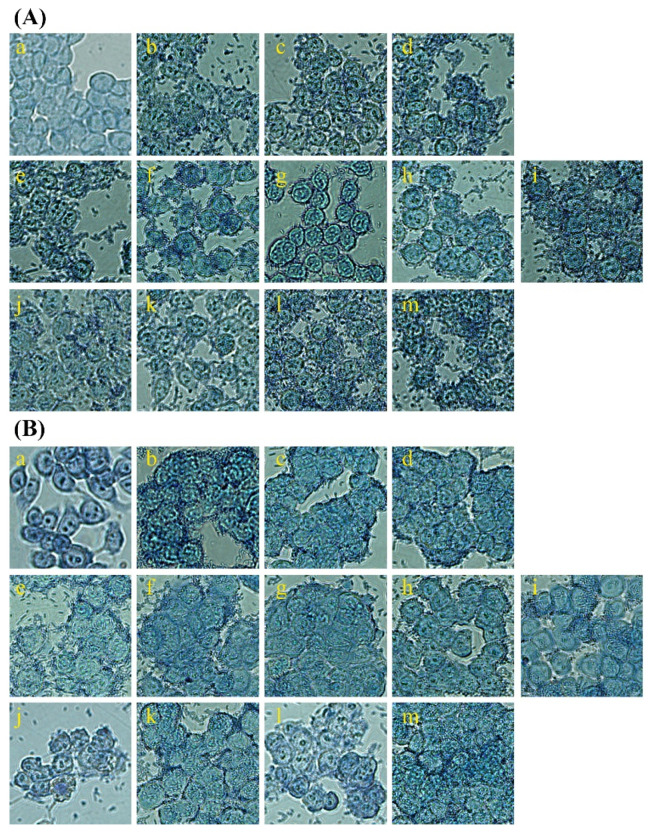
Adherence of strains to Caco-2 and HT-29 cells visualized through Giemsa staining (magnification: 800×). (**A**) was confirmed to have strain adhesion ability to Caco-2 cells and (**B**) to HT-29 cells. a: Blank control (no bacterial treatment); b: *L. paracasei* (type strain); c: *S. thermophilus* (type strain); d: *L. plantarum* (type strain); e: JAMI_LB_01; f: JAMI_LB_02; g: JAMI_LB_03; h: JAMI_LB_04; i: JAMI_LB_05; j: JAMI_LB_01 + JAMI_LB_03; k: JAMI_LB_01 + JAMI_LB_05; l: JAMI_LB_02 + JAMI_LB_03; m: JAMI_LB_02 + JAMI_LB_05.

To further assess the ability of LAB to adhere to the intestinal mucosa, Giemsa staining was performed, and the results were visualized under a microscope (Figs. 4). Compared to blank controls, all LAB treatment groups displayed rod-shaped bacteria adhering to the cell surface. Panels (b)–(d) of Figure 4 represent the standard strains (*L. paracasei*, *S. thermophilus*, and *L. plantarum*), which are recognized as probiotic materials. These strains demonstrated adhesion to the cell surface according to treatment. Panels (e)–(i), corresponding to LAB strains isolated from fermented milk and beverages, also confirmed adhesion to the cell surface. Among these, the JAMI_LB_05 strain exhibited the highest density of attached LAB compared to other strains.

## 4. Discussion

This study investigated the probiotic potential of five LAB strains isolated from fermented milk and soymilk products. The evaluation focused on their survival in harsh gastrointestinal conditions, adhesion to intestinal epithelial cells, and functional activities to identify strains with promising probiotic properties. For probiotics to exert their physiological benefits in the human body, they must demonstrate high survival rates in stomach acid and bile and possess antibacterial properties to inhibit harmful bacteria [7]. The probiotic characteristics of microorganisms, including acid resistance, bile resistance, and antibiotic resistance, vary depending on the strain and can differ even among strains within the same species [26,27].

The survival of the LAB strains in artificial gastric and intestinal fluids was strain-dependent. In the gastric fluid model, the *L. plantarum* strains exhibited survival rates exceeding 90%, while in the bile fluid model, the *S. thermophilus* strain demonstrated notable survival rates. A previous study on LAB isolated from wine reported that a reference *L. plantarum* strain maintained a viable bacterial count of 7.95 ± 0.06 log CFU/mL under pH 2.1 conditions [7]. In comparison, the strains JAMI_LB_03, JAMI_LB_04, and JAMI_LB_05 from our study showed viable bacterial counts of 8.62 ± 0.42 log CFU/mL, 10.02 ± 0.37 log CFU/mL, and 9.82 ± 0.42 log CFU/mL, respectively, confirming superior acid resistance. These results suggest that the strains are highly adaptable to the intestinal environment, surpassing some species’ standard acid and bile resistance.

In general, concerns have been raised about the potential horizontal gene transfer of antibiotic resistance genes to pathogenic bacteria when using certain probiotic strains with antibiotic resistance genes. However, antibiotic resistance can enhance the intestinal mucosal adhesion of LAB following antibiotic treatment [28]. The LAB isolated from fermented milk exhibit varying degrees of antibiotic resistance or susceptibility, depending on the type of LAB. To determine antibiotic resistance, phenotypes are typically confirmed using the minimum inhibitory concentration (MIC) evaluation method, while antibiotic resistance genes are identified using PCR techniques and microarrays. In this study, only phenotypes were confirmed using the MIC evaluation method, which lacks consistency and is a limitation. Separately, *Enterococcus faecalis* and *Enterococcus faecium* can only be used in Korea if they lack antibiotic resistance and toxic genes [29]. Identifying antibiotic resistance genes in the nucleotide sequences of LAB will facilitate compliance with regulations when determining endogenous resistance. The LAB isolated and used in this study did not contain *Enterococcus*, and further in-depth studies on antibiotic resistance are needed in future research.

For LAB to function as effective probiotics, they must survive passage through the stomach and duodenum, reach the intestines, and adhere to intestinal epithelial cells [30]. In this study, the adherence of the isolated strains was evaluated using Caco-2 and HT-29 intestinal epithelial cells. When applied individually, the adhesion rates of the five LAB strains were below 6%. However, mixtures of two strains resulted in a significant increase in adhesion ability, highlighting a synergistic effect. This result aligns with previous findings emphasizing the benefits of using mixed probiotics on Caco-2 and HT-29 cells [17,24,31]. The LAB strains adhered to Caco-2 cells and inhibited the secretion of inflammatory cytokines, such as IL-6 and IL-8, suggesting that the LAB strains did not induce an inflammatory response in human intestinal cells [32]. As the understanding of probiotics advances, the importance of developing blended formulations has become increasingly evident. Single-strain probiotics are unlikely to colonize the gut comprehensively or achieve all desired therapeutic effects. Mixed probiotics can target multiple conditions and optimize efficacy [15]. The adhesion of probiotics allows for their colonization of the human intestine, and although transient [33], it is associated with several health benefits attributed to probiotics. A mixture of strains (*L. rhamnosus* GG, *L. rhamnosus* LC705, *B. breve* 99 and *P. freudenreichii* ssp.) reduced the adhesion of tested pathogen strains to intestinal mucus through inhibition, displacement, and competition mechanisms [34]. Inhibition against some pathogen infections was induced by more than 40%. These characteristics suggest that LAB with antibiotic resistance may effectively inhibit pathogen attachment.

LAB adherence to the intestinal mucosa is critical in suppressing harmful bacterial proliferation by competing for nutrients and attachment sites. This competitive exclusion helps maintain a balanced intestinal environment. LAB surface hydrophobicity, auto-aggregation, and co-aggregation contribute to their adhesion properties [35]. For instance, LAB strains can co-aggregate with other bacterial cells or mucus components, enhancing their ability to adhere to the intestinal epithelium or mucus layer. Additionally, LAB may interact with extracellular matrix molecules such as collagens, fibronectin, and vitronectin, which can facilitate adhesion [36]. Studies show that certain probiotic bacterial strains exert anti-inflammatory effects by the ability to favor the induction of IL-10-dependent, TGF-b-bearing regulatory cells [37,38,39]. Efforts are underway to improve microbiota composition using probiotics with strong adhesion in the body. Studies continue to show that gut microbiota and intestinal barrier function affect chronic inflammatory and autoimmune diseases [38,40,41,42]. The multi-strain LAB formulations tested in this study demonstrated enhanced adhesion ability, supporting their potential use as functional probiotic materials. Microorganisms with strong adhesive properties are better equipped to colonize the gut and displace pathogenic bacteria, contributing to gut health [43]. Based on findings, the LAB mixtures identified in this study could be investigated further for properties such as surface hydrophobicity and auto-aggregation to enhance their probiotic applications.

This study primarily assessed probiotic activity in an in vitro model, and a limitation is in replicating the complexities of the actual intestinal environment. In vivo studies are required to validate these findings and optimize strain combinations for clinical applications. Future research should focus on using in vivo models to confirm the colonization potential of mixed LAB strains and optimize strain combinations and ratios. Nevertheless, LAB strains with high adhesion ability hold significant promise for use in dairy products and health supplements. These findings underline the potential of LAB as a functional food component to support gut health and overall well-being.

## 5. Conclusions

In summary, this study successfully isolated and evaluated Lactobacillus strains from fermented milk and beverages to identify potential probiotics for human applications. The strains, identified as *L. paracasei*, *S. thermophilus*, and *L. plantarum*, exhibited strong probiotic properties, including survival rates exceeding 90% in artificial gastric juice and over 70% in artificial bile juice, demonstrating their resilience in the gastrointestinal environment. The LAB strains showed significant adhesion abilities to human intestinal epithelial cell lines, with a notable enhancement in adhesion when strains were used in combination. Specifically, strain JAMI_LB_05 displayed both strong intestinal adhesion and high antibiotic resistance. When combined with JAMI_LB_02, the mixed strains exhibited a synergistic improvement in adhesion, suggesting their potential for enhanced colonization and functional activity in the intestine. These findings highlight the probiotic potential of the isolated LAB strains, particularly in mixed formulations, which could offer improved intestinal survival, adaptability, and functional efficacy. Further studies, particularly in vivo, are warranted to validate their clinical applications and optimize strain combinations for human health benefits.

## 6. Patents

The strain utilized in this study has been registered under a Republic of Korea patent (Patent No. 10-2024-0167216).

## Figures and Tables

**Table 1:** Identification of bacterial strains by sequencing the universal 16S rRNA region.

Strain Name	Source	Accession No. of Strain	Species Identification Based on 16S Sequence	E-Value	Similarity (%) of 16S Sequence to That of Reference Strains in GenBank
JAMI_LB_01	Fermented soy milk	AP012541.1	*Lacticaseibacillus paracasei*	0.0	99.80
JAMI_LB_02	Fermented soy milk	NR_042778.1	*Streptococcus thermophilus*	0.0	99.73
JAMI_LB_03	Filtered fermented milk	NR_104573.1	*Lactiplantibacillus plantarum*	0.0	99.86
JAMI_LB_04	Fermented milk	NR_104573.1	*Lactiplantibacillus plantarum*	0.0	99.93
JAMI_LB_05	Fermented milk	NR_104573.1	*Lactiplantibacillus plantarum*	0.0	99.93

**Table 2:** Survival rates of selected strains in artificial gastric and intestinal juice.

Strain Name	Control (log CFU/mL)	Gastric Juice (log CFU/mL)	Survival (%)	Intestinal Juice (log CFU/mL)	Survival (%)
JAMI_LB_01	8.95 ± 0.07 ^1^	5.16 ± 0.00	57.67 ± 0.03 ^b1^	4.54 ± 0.76	50.71 ± 8.52 ^a^
JAMI_LB_02	8.80 ± 0.71	3.72 ± 0.17	42.31 ± 1.95 ^a^	7.67 ± 0.00	87.17 ± 0.02 ^e^
JAMI_LB_03	9.50 ± 0.04	8.62 ± 0.42	90.70 ± 4.45 ^d^	7.37 ± 0.31	77.51 ± 3.26 ^de^
JAMI_LB_04	10.46 ± 0.20	10.02 ± 0.37	95.73 ± 3.52 ^d^	7.81 ± 0.33	74.60 ± 3.11 ^cd^
JAMI_LB_05	10.15 ± 0.27	9.82 ± 0.42	96.71 ± 4.10 ^d^	7.96 ± 0.45	78.36 ± 4.44 ^de^
JAMI_LB_01 + JAMI_LB_03	9.98 ± 0.67	7.76 ± 0.21	77.76 ± 2.06 ^c^	7.03 ± 0.35	70.45 ± 3.55 ^bcd^
JAMI_LB_01 + JAMI_LB_05	9.94 ± 0.29	7.10 ± 0.71	71.43 ± 7.11 ^c^	6.00 ± 0.43	60.34 ± 4.28 ^ab^
JAMI_LB_02 + JAMI_LB_03	9.24 ± 0.09	6.80 ± 0.71	73.62 ± 7.65 ^c^	7.13 ± 0.21	77.21 ± 2.23 ^de^
JAMI_LB_02 + JAMI_LB_05	9.50 ± 0.04	6.98 ± 0.71	73.41 ± 7.44 ^c^	6.25 ± 0.49	65.79 ± 5.20 ^bc^

^1^ Values are presented as means ± SD (n = 2). Different lowercase letters (e.g., a, b, c, d, e) within the same row indicate significant differences, as determined by Duncan’s multiple range test (*p* < 0.05).

**Table 3:** Minimum inhibitory concentration of antibiotics for the probiotic strains.

Species	Strain	MIC (μg/mL)				
		AMP ^1^	TET	ERY	KAN	STR
*L. paracasei*	JAMI_LB_01	16	64	2	1024	1024
*S. thermophilus*	JAMI_LB_02	2	4	2	1024	128
*L. plantarum*	JAMI_LB_03	2	123	8	1024	1024
*L. plantarum*	JAMI_LB_04	8	64	4	1024	1024
*L. plantarum*	JAMI_LB_05	4	256	8	1024	1024
Microbiological cut-off value (mg/L) proposed by EFSA						
*L. paracasei*		4	4	1	64	64
*S. thermophilus*		2	4	2	64	64
*L. plantarum*		2	32	1	64	NR ^2^

^1^ AMP, TET, ERY, KAN, and STR refer to ampicillin, tetracycline, erythromycin, kanamycin, and streptomycin. ^2^ NR, not required.

## Data Availability

The bacterial strains analyzed in this study are deposited in the Korean Agricultural Culture Collection (KACC), Republic of Korea, under accession numbers KACC 92623P and KACC 92624P.
